# Resveratrol Modulates Transforming Growth Factor-Beta (TGF-β) Signaling Pathway for Disease Therapy: A New Insight into Its Pharmacological Activities

**DOI:** 10.3390/biomedicines8080261

**Published:** 2020-07-31

**Authors:** Milad Ashrafizadeh, Masoud Najafi, Sima Orouei, Amirhossein Zabolian, Hossein Saleki, Negar Azami, Negin Sharifi, Kiavash Hushmandi, Ali Zarrabi, Kwang Seok Ahn

**Affiliations:** 1 Department of Basic Science, Faculty of Veterinary Medicine, University of Tabriz, Tabriz 5166616471, Iran; dvm.milad73@yahoo.com; 2 Radiology and Nuclear Medicine Department, School of Paramedical Sciences, Kermanshah University of Medical Sciences, Kermanshah 6715847141, Iran; najafi_ma@yahoo.com; 3 Department of Genetics, Tehran Medical Sciences, Islamic Azad University, Tehran 1916893813, Iran; Sima.orouei@gmail.com; 4 Young Researchers and Elite Club, Tehran Medical Sciences, Islamic Azad University, Tehran 1916893813, Iran; Fzr2000_0007@yahoo.com (A.Z.); hosseinsaleki2015@gmail.com (H.S.); negarazami77@gmail.com (N.A.); Negin.sharifi87@gmail.com (N.S.); 5 Department of Food Hygiene and Quality Control, Division of Epidemiology & Zoonoses, Faculty of Veterinary Medicine, University of Tehran, Tehran 1417414418, Iran; houshmandi.kia7@ut.ac.ir; 6 Sabanci University Nanotechnology Research and Application Center (SUNUM), Tuzla, 34956 Istanbul, Turkey; 7 Center of Excellence for Functional Surfaces and Interfaces (EFSUN), Faculty of Engineering and Natural Sciences, Sabanci University, Tuzla, 34956 Istanbul, Turkey; 8 Department of Science in Korean Medicine, College of Korean Medicine, Kyung Hee University, 24 Kyungheedae-ro, Dongdaemun-gu, Seoul 02447, Korea

**Keywords:** resveratrol, transforming growth factor-beta (TGF-β), chronic diseases, fibrosis, cancer, diabetes, therapy

## Abstract

Resveratrol (Res) is a well-known natural product that can exhibit important pharmacological activities such as antioxidant, anti-diabetes, anti-tumor, and anti-inflammatory. An evaluation of its therapeutic effects demonstrates that this naturally occurring bioactive compound can target different molecular pathways to exert its pharmacological actions. Transforming growth factor-beta (TGF-β) is an important molecular pathway that is capable of regulating different cellular mechanisms such as proliferation, migration, and angiogenesis. TGF-β has been reported to be involved in the development of disorders such as diabetes, cancer, inflammatory disorders, fibrosis, cardiovascular disorders, etc. In the present review, the relationship between Res and TGF-β has been investigated. It was noticed that Res can inhibit TGF-β to suppress the proliferation and migration of cancer cells. In addition, Res can improve fibrosis by reducing inflammation via promoting TGF-β down-regulation. Res has been reported to be also beneficial in the amelioration of diabetic complications via targeting the TGF-β signaling pathway. These topics are discussed in detail in this review to shed light on the protective effects of Res mediated via the modulation of TGF-β signaling.

## 1. Resveratrol

From immemorial times, plant-derived natural compounds have been under attention in the treatment of different disorders such as inflammatory diseases, cancers, pulmonary diseases, metabolic disorders, neurological disorders (NDs) including Alzheimer’s disease (AD) and Parkinson’s disease (PD), infertility, and so on [1,2,3,4,5,6,7,8,9,10]. Phytochemicals can exhibit beneficial actions against diseases due to their excellent pharmacological activities [11,12,13,14]. These benefits have resulted in extensive research into finding new natural compounds and revealing their potential mechanisms of actions [15,16,17]. Resveratrol (Res) is a dietary phytochemical that has been reported to be efficacious treatment for various ailments by targeting diverse molecular pathways [18,19,20,21]. The role of Res in the treatment of chronic diseases was established in early 1990s when it was found that this phytochemical possesses significant cardioprotective benefits [22]. This ascending trend toward Res research led to the revelation of its significant biological and therapeutic activities. The first report about anti-tumor activity of Res dates back to 1997, when Jang and his colleagues reported its inhibitory effect on leukemia [23].

Currently, Res can be derived from various plants including *Arachis hypogea*, *Cassia* sp., *Eucalyptus* sp., *Morus rubra*, and so on using a number of different isolation techniques [24]. High-performance liquid chromatography is the best strategy [25,26,27,28]. Over the past decades, Res has been applied in the treatment of various diseases such as osteoarthritis [29,30,31], NDs [32], cancer [33,34,35], diabetes [36], cardiovascular diseases [37], liver disorders [38], and so on. An increasing amount of evidence is in agreement with the fact that Res affects different molecular pathways to exhibit its protective effects [39,40,41]. Hence, the identification of these targets can promote further studies for investigating molecular pathways and the mechanisms of its therapeutic actions in depth. For instance, anti-inflammation is one of the most important biological effects of Res treatment. To function as an anti-inflammatory molecule, Res can effectively inhibit the activation of pro-inflammatory transcription factors such as nuclear factor-kappaB (NF-ĸB). It seems that the anti-inflammatory actions of Res are not only mediated via inhibitory actions on the NF-ĸB signaling pathway, but they also rely on its action as a PARP-γ agonist [42]. The anti-inflammatory activities of Res are also characterized by decreased levels of interleukin (IL)-6, IL-8, and tumor necrosis factor-α (TNF-α), etc. [43]. The production of pro-inflammatory lipid mediators from arachidonic acid can be mediated by the cyclooxygenase (COX) pathway. A number of anti-inflammatory drugs have been developed based on their inhibitory effect on COX-1 and COX-2 [44,45]. Res is capable of binding to the active site of COX-1 and thus causing anti-inflammatory effects. In addition to targeting inflammation, Res attaches to the active site of COX-2 to suppress cancer proliferation [46,47,48,49]. It is noteworthy that the inhibitory effect of Res on COX has been noted to follow a dose-dependent kinetics [50].

Obesity is one of the challenges faced in today’s world. Res has demonstrated great potential in reducing weight and exerting anti-obesity activity. Res changes white adipose tissue (WAT) into brown adipose tissue (BAT), which in turn decreases weight and improves insulin resistance [51]. The inhibitory action of Res on lipid accumulation leads to its effect on cardiovascular disorders. Res stimulates PARP-α/γ to activate ATP binding cassette (ABC) transporter A1/G1-mediated cholesterol efflux, resulting in a decrease in lipid accumulation and cholesterol levels. These effects can lead to a significant amelioration of atherosclerosis [52]. Based on the effect of Res on amyloid-beta (Aβ), this plant-derived natural compound is of importance in treating NDs. For instance, Res is able to inhibit inflammation and the microglial activation caused by Aβ. This results in the alleviation of inflammation (down-regulation of TNF-α and IL-6) and a diminution in apoptosis (caspase-1 down-regulation) [53]. The antioxidant activity of Res provides its protective effect during kidney injury. In rats exposed to nicotine, an increase occurs in oxidative stress markers via the down-regulation of glutathione. The administration of Res has been also correlated with improving the antioxidant defense system that protects renal cells against oxidative injury [54]. A newly published study also demonstrates the effect of Res on stem cells. Res can stimulate stem cell function to ameliorate pancreatic injury such as fibrosis and apoptosis [55]. Overall, these reports exhibit that Res has diverse therapeutic effects that have resulted in its extensive application in the treatment of various disorders [56,57,58]. In the current review, we specifically focus on the therapeutic effects of Res mediated by its regulatory action on the transforming growth factor-β (TGF-β) signaling pathway.

### 1.1. Resveratrol: Limitations and Applied Strategies

In spite of the excellent pharmacological activities of plant-derived natural compounds, very soon it was found that a number of issues limit their efficacy in disease treatment. Increasing evidence shows that phytochemicals are able to exert their therapeutic effects predominantly under in vitro settings. However, when their efficiency is examined for in vivo experiments, a decrease occurs in their therapeutic efficacy due to their potential poor bioavailability. The difficulty is more prominent in clinical trials, leading to a limited application of phytochemicals in clinic. This holds also true for Res, and various formulations of this agent have been tested to enhance its therapeutic capabilities. Res has a lipophilic nature and can be dissolved in fruit or vegetable juices or given in capsule form. The administration frequency of Res is variable from one to three times a day, and its reported doses are at the range of 0.073 mg to 5 g [59,60]. The reports also demonstrated that the most efficient strategy in promoting the bioavailability and protective effects of Res is using nanoparticles [61]. The encapsulation of Res by nanoparticles protects against degradation and improves its intestinal absorption and blood circulation time [62,63,64,65,66]. These benefits lead to the promoted bioavailability of Res and an improvement in its therapeutic effects [67,68]. It has been reported that loading Res on lipid carriers can significantly increase its anti-tumor activity and cytotoxicity against breast cancer cells by providing targeted delivery and enhancing its intracellular internalization [69]. Lipid nanocarriers containing Res can be administered through the oral route. The oral administration of Res-loaded lipid nanostructures is more beneficial in reducing the levels of pro-inflammatory cytokines and induction of anti-inflammatory activity compared to Res alone [70]. The enhanced release of Res in the intestine by nanoparticles is of importance in elevating its cytotoxicity against cancer cells [71]. Overall, various studies reveal that nanostructures can be considered as potential delivery systems for Res and fortunately, a significant number of studies have been performed in this field. The findings are in line with the fact that these nano-based strategies can remarkably enhance both the bioavailability and therapeutic capability of Res [72,73,74]. However, more studies are needed to design different effective nanocarriers to facilitate an optimum delivery of Res.

### 1.2. Pharmacokinetics of Resveratrol: A Brief Explanation

Increasing evidence demonstrates that the dosage forms and conditions of patients can affect the absorption of Res. However, the gastrointestinal (GI) tract is involved in the absorption of Res after oral administration with a peak at plasma concentration after 30 min and 1.5–2 h [75,76,77]. The absorption of Res undergoes an increase via grape consumption and using other forms such as micronized form [78,79,80,81,82]. After absorption, Res can be distributed in different organs, such as the brain, liver, intestine, and fat [83]. For metabolism, enterocytes and hepatocytes play the most important role after oral administration. Notably, Res influx occurs through the passive diffusion and carrier-mediated process [84,85]. The metabolism of Res also confirms its distribution in liver, so that it has been reported that Res is a substrate of hepatic sulfotransferase and glucuronosyltransferase, and it extensively accumulates in liver [86]. The interesting point is that metabolism of Res relies on dose. Low doses (5–50 mg) of Res are bio-transformed into glucuronides, while high doses (more than 250 mg) are bio-transformed into monosulfates [87,88,89,90,91]. Facial areas and urine are responsible for the elimination of Res. It has been noted that the administration form of Res may affect its elimination, which can be delayed when micronized Res is used [92,93,94,95,96].

### 1.3. Toxicity of Resveratrol

Similar to other compounds, plant-derived natural compounds have a number of drawbacks. Although Res is safe and well-tolerated at normal doses, there are toxicities associated with the application of high doses of Res [97]. The willingness toward using high doses of Res is due to its poor bioavailability, which restricts its therapeutic usage. Therefore, providing information about the toxicity of Res is advantageous for directing further studies toward using normal and safe doses of Res. It is worth mentioning that the toxicity of Res has been evaluated in both in vivo and clinical trials. It appears that high doses of Res—as much as 3 g/kg/day in rats—may result in nephrotoxicity. Although there are few studies that have demonstrated that Res can negatively affect liver and enhance levels of liver enzymes such as aspartate aminotransferase, others have reported that it may not exhibit any significant toxicity on the liver [98,99]. The administration of 750 mg/kg/day of Res for 3 months is well-tolerated in rats [100]. Studies in humans show that Res is completely safe and only a few adverse effects including blood electrolyte changes, nasopharyngitis, and erythematous rash can be observed after the administration of 400 mg of Res. Headache, myalgia, epididymitis, and dizziness were other commonly reported adverse effects of Res [101,102,103].

## 2. TGF-β: Signaling Pathways and Pathological Role

### 2.1. Members and Receptors of TGF-β Family

There are three distinct members of TGF-β in mammals including TGF-β1, TGF-β2, and TGF-β3 that are homologous in terms of structure, but they demonstrate different biological activities, temporal, and spatial expression patterns [104,105,106,107,108]. The number of genes that can encode members of the TGF-β family are numerous, but a number of them can be mentioned as *activin*, *nodal*, *bone morphogenetic proteins* (*BMPs*), and *growth and differentiation factors* (*GDFs*) [109]. The TGF-β signaling pathway possesses a regulatory effect on different cellular events such as growth, survival, differentiation, cell fate specification, angiogenesis, and so on [110,111,112,113,114,115]. TGF-β signaling is initiated by the attachment of a ligand onto cell surface receptors, which in turn triggers a cascade that mediates the translocation of TGF-β into the nucleus. In humans, there are 12 cell surface receptors that are affected by ligand, including type I receptors (ALK1-7) and type II receptors (TβRII, ActRII, ActRIIB, BMPRII, and AMHRII) [116,117]. After attachment of a certain type of TGF-β into type II receptors, these receptors are stimulated, which subsequently phosphorylates the glycine-serine-rich domain (GS domain) of type I receptors. In the canonical pathway of TGF-β, type I receptors mediate the formation of Smad complex via phosphorylation at carboxyl termini.

### 2.2. TGF-β Signaling Pathway

**Figure 1:**
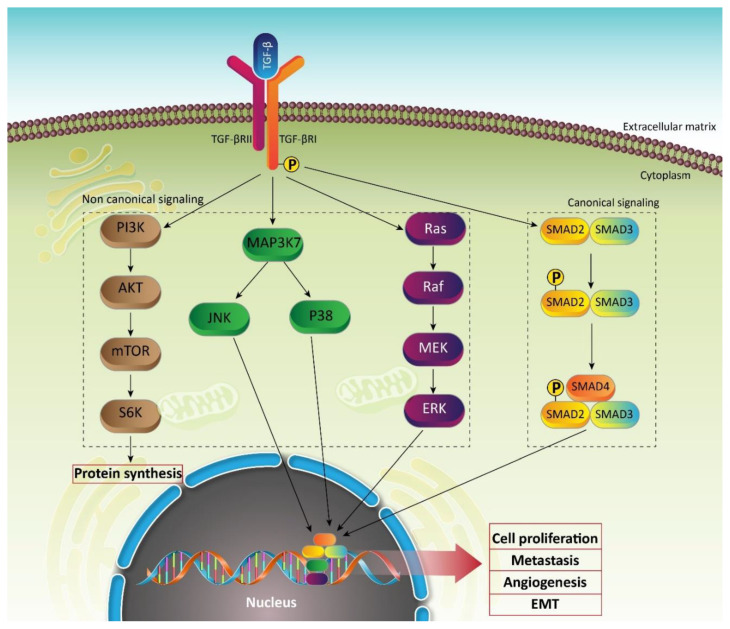
A schematic presentation of transforming growth factor-beta (TGF-β) signaling pathways. This pathway consists of two distinct modules: canonical signaling and non-canonical signaling. Canonical signaling, as shown in the figure, is a result of the formation of a complex containing Smad2, Smad3, and Smad4. Then, these molecules can translocate into the nucleus to trigger the expression of genes that are responsible for the proliferation and metastasis of cancer cells. Non-canonical signaling is Smad-independent and involves different signaling pathways such as PI3K/Akt, MAP3K7, Ras, and so on. However, final aim of these two signaling pathways is to promote aberrant growth and malignancy of cancer cells.

The *TGF-β* gene encodes a pro-precursor peptide consisting of 390 amino acids that undergoes proteolytic processing to produce mature TGF-β. This mature TGF-β has two distinct sections including amino-terminal and carboxy-terminal sections [118]. The amino-terminal fragment is known as latency associated peptide (LAP) with non-covalent attachment into TGF-β [119,120]. The cleavage of LAP by proteases or mechanical forces by cell surface integrins contributes to the release of mature and active TGF-β [121,122]. The activated TGF-β is a dimeric protein with disulfide bonds and molecular weight of 25 kDa that can bind into cell surface receptors. As described above, then, the binding of a ligand into a receptor leads to the phosphorylation of type I receptors by type II ones [123]. Then, TGF-βRI as a type I receptor can stimulate Smad2 and Smad3 via phosphorylation, resulting in the formation of a complex with Smad4. This complex translocates into the nucleus to affect target genes such as *plasminogen activator inhibitor 1* (*PAI*1). Among them, only Smad4 and Smad3 can bind to DNA. It is worth mentioning that the affinity of Smad3 and Smad4 for attachment to DNA is low and they need to collaborate with other DNA-binding transcription factors to promote gene expression [124,125]. This is the canonical pathway of TGF-β, and there is another pathway, which is known as the non-canonical pathway. In this pathway, activated receptors target different molecular pathways such as PI3K as well as JNK, P38, extracellular signal-regulated kinase (ERK), and mitogen-activated protein kinase (MAPK). For instance, PI3K can be activated by stimulated receptors to induce Akt/mTOR axis, resulting in the stimulation of S6K and regulate protein translation (Figure 1) [106].

### 2.3. TGF-β in Cancer, Diabetes, and Other Pathological Events

A number of studies have highlighted that the abnormal expression of TGF-β may pave the road for generating pathological events. The role of the TGF-β signaling pathway in cancer cells has been extensively investigated. Increasing evidence demonstrates that TGF-β mediates the migration and invasion of cancer cells. For enhancing cancer cell metastasis, TGF-β induces epithelial-to-mesenchymal transition (EMT), which significantly promotes the migratory ability of cancer cells [126]. Interestingly, molecular pathways that negatively regulate the metastasis of cancer cells can reduce the expression of TGF-β. It has been revealed that sirtuin 7 (SIRT7) can suppress the migration of cancer cells through inhibiting TGF-β signaling via Smad4 degradation. Therefore, the Smad complex may be disrupted, and its nuclear translocation can be inhibited [127]. In addition to metastasis, TGF-β signaling induces angiogenesis, which is a mechanism that is vital for the proliferation and migration of cancer cells. The stimulatory effect of TGF-β on angiogenesis can be mediated via the phosphorylation of Smad3 [128]. TGF-β is able to stabilize the Nrf2 signaling pathway via p21 induction, thus leading to the chemoresistance of cancer cells [129]. Moreover, numerous studies are in agreement with the fact that TGF-β can act as a positive factor for the proliferation and migration of cancer cells, and a negative factor for cancer prognosis. In addition to cancer, TGF-β contributes to the development of other malignancies. Diabetes mellitus (DM) is a chronic metabolic disorder in which insulin resistance can be obtained and glucose metabolism undergoes dysregulation [130,131]. Myocardial injury and fibrosis may result from DM, and studies have demonstrated that TGF-β is involved in this process. In DM, TGF-β activates Smad2 to facilitate its nuclear translocation. Then, an increase occurs in fibrosis, thereby providing conditions for deteriorating DM. Mesenchymal stem cell-derived exosomes are able to improve DM fibrosis via the inhibition of the TGF-β/Smad2 axis [132]. The TGF-β/Smad3 axis may be also involved in DM fibrosis. Thus, the stimulation of TGF-β and the nuclear translocation of Smad3 provide conditions for the development of renal fibrosis during DM. It has been found that the administration of retinoic acid can alleviate DM-promoted fibrosis via the inhibition of TGF-β/Smad3 [133]. It is noteworthy that a number of phytochemicals have shown potential in the regulation of the TGF-β signaling pathway, which is of immense importance for disease therapy [134,135]. In the present review, we focus on modulation of the TGF-β signaling pathway by Res and its potential impact for disease therapy [136,137,138].

## 3. Resveratrol and TGF-β Signaling Pathway

In this section, we will highlight the modulatory effects of Res on TGF-β levels in different chronic diseases. For example, Res can suppress the TGF-β signaling pathway and its downstream targets such as Smads. It can also reduce TGF-β-mediated EMT in fibrosis. It has been reported that for the inhibition of EMT, Res can down-regulate matrix metalloproteinase-9 (MMP-9), leading to the alleviation of fibrosis. MicroRNAs (miRs) such as miR-31 can also be affected by Res in targeting TGF-β in disease therapy. The inhibitory effect of Res on the TGF-β signaling pathway can lead to the suppression of intra-abdominal adhesion formation, since TGF-β can enhance fibrin accumulation [139,140,141,142,143,144,145,146,147,148,149]. These modulatory effects of Res are discussed in the following sections.

### 3.1. Resveratrol and Fibrosis

Pulmonary fibrosis (PF) is a common disorder of the lung that is characterized with hypoxemia, restrictive functional ventilatory disturbance, and chronic fibrosis. Clinical manifestations of PF include wheezing, difficulties in breathing, and dry coughs [150]. The pathogenesis of PF is still not completely understood, but it appears that the TGF-β signaling pathway plays a significant role in PF development [151]. Thus, the administration of Res may be an ideal strategy in the amelioration of PF, and different molecular pathways may be involved. Normally, microRNA (miR)-21 can induce PF via the activation of TGF-β signaling and providing Smad7 nuclear translocation. TGF-β provides a positive feedback loop, so TGF-β enhances the expression of miR-21 and AP-1. The administration of Res down-regulates the expression of miR-21 via inhibition of the MAPK/AP-1 axis. This leads to a diminution in TGF-β expression and inhibition of Smad7, resulting in the alleviation of PF [152]. Accumulating data demonstrate that during the inhibition of fibrosis, Res affects the TGF-β signaling pathway via the modulation of miRs. Myocardial fibrosis (MF) is caused by the accumulation of collagen fibers, enhanced collagen content, and alteration in collagen composition. Systolic and diastolic functions of the heart can be negatively affected by MF [153]. TGF-β is one of the key players regulating MF [154]. The TGF-β/Smad7 axis can also contribute to the development of MF. The administration of Res can up-regulate the expression of miR-17, which in turn remarkably reduces levels of Smad7, leading to an improvement in MF [155].

In addition to PF and MF, renal fibrosis (RF) can arise as a result of the activation of the TGF-β signaling pathway. It has been reported that the inhibition of the TGF-β signaling pathway by natural products such as bardoxolone and nimbolide is of importance in RF therapy [156,157]. It is worth mentioning that Res can target the TGF-β signaling pathway, thereby causing an amelioration of RF. In RF treatment, fibroblast–myofibroblast differentiation (FMD), EMT, and the proliferation of tubular epithelial cells (TECs) should be targeted. The administration of Res can disrupt Smad2/3 activation by TGF-β and consequently suppress the proliferation of TECs, FMD, and EMT [158]. Increasing evidence demonstrates that EMT may be involved in renal fibrogenesis, and its activation can facilitate the development of RF [159,160,161,162]. Res is capable of suppressing EMT-mediated RF. It seems that TGF-β1 functions as an upstream mediator of EMT, and Res suppresses EMT and RF through inhibiting TGF-β1 [163]. In fact, in the stimulation of anti-fibrotic activity, Res affects the proliferation and survival of fibroblasts. It has been shown that Res can stimulate apoptosis in fibroblasts and suppress their growth as well. An investigation of the molecular pathways demonstrates that in targeting fibroblasts, Res can suppress TGF-β and the Smad2/3/4 complex, and it can also upregulate Smad7 [164].

It is worth mentioning that the anti-fibrotic activity of Res is dose-dependent, and using low doses is preferred as compared to higher doses. An experiment has evaluated the role of dose in the anti-fibrotic activity of Res. TGF-β induces fibrosis via formation of the Smad3/4 complex and subsequent stimulation of EMT. The administration of Res has been correlated with the deacetylation of Smad3 and Smad4 via sirtuin 1 (SIRT1). According to in vitro results, low doses of Res (5–20 mM) effectively exerted anti-fibrotic activity, while high doses (more than 40 mM) did not demonstrate any substantial anti-fibrotic activity. The in vivo findings are in line with in vitro results, so that low doses of Res (less than 25 mg/kg) improve fibrosis, while high doses of Res (more than 50 mg/kg) deteriorated the condition [165]. This study confirms the dose-related toxicity of Res. Overall, these studies demonstrate that TGF-β can function as a key player in the development of fibrosis and Res can suppress the TGF-β signaling pathway and its downstream targets such as Smads to alleviate fibrosis [166,167].

The TGF-β signaling pathway contributes to the development of fibrosis in different vital organs of body such as the lung and heart. The interesting point to highlight is the possible epigenetic regulation of TGF-β by miRs in the development of fibrosis. Res is capable of suppressing miR and TGF-β interaction in fibrosis therapy. MiR-17 and miR-21 are two important miRs that contribute to the emergence of myocardial and pulmonary fibrosis via TGF-β induction. The regulation of TGF-β by miRs is suppressed upon Res administration. RF also occurs by the function of TGF-β and subsequent induction of EMT. The TGF-β/EMT axis is inhibited by Res to alleviate RF. It is noteworthy that in the amelioration of fibrosis, components of TGF-β signaling such as Smad7 and Smad4 can also be down-regulated. Therefore, TGF-β is a versatile agent in the amelioration of fibrosis.

### 3.2. Resveratrol and Cancer Therapy

Accumulating data exhibit that the TGF-β signaling pathway can regulate both the proliferation and metastasis of cancer cells, and its inhibition is a promising strategy in cancer therapy [168,169,170,171,172,173]. Metastasis is an increasing challenge in the effective treatment of cancer. Cancer cells are able to migrate into neighboring and distant tissues, demanding novel strategies in the inhibition of their metastasis. EMT is one of the mechanisms that can promote invasion via the transformation of static epithelial cells into migratory mesenchymal ones [174]. A number of different molecular pathways have been recognized as regulators of EMT [175,176], and it has been found that TGF-β is capable of elevating migration via EMT induction. In breast cancer, TGF-β can stimulate EMT via Smad2 and Smad3 activation, leading to an increase in N-cadherin and vimentin levels, and a decrease in E-cadherin levels. The administration of Res suppresses the metastasis of breast cancer (under both in vitro and in vivo conditions) via the inhibition of TGF-β1 and down-regulation of Smad2 and Smad3 [177]. TGF-β also contributes to the migration and malignant behavior of lung cancer. In addition to breast cancer, Res targets TGF-β to inhibit EMT in lung cancer. By suppressing levels of TGF-β, Res down-regulates the levels of vimentin and fibronectin, while it enhances E-cadherin levels, leading to an inhibition of EMT and metastasis of lung cancer cells [178]. It is noteworthy that EMT induction enhances viability via the stimulation of cancer stem cell markers such as Bmi1 and Sox2. By inhibition of the TGF-β/Smad axis, Res not only inhibits EMT and migration, but also interferes with the proliferation and survival of cancer cells [179]. So, Res can function as a potential modulator of EMT in cancer cells to negatively affect their proliferation and metastasis.

Accumulating data also show that Res is able to diminish levels of TGF-β that in turn, suppresses the development of renal carcinoma [180]. These studies are in agreement with the fact that the inhibition of TGF-β by Res is of interest in suppressing tumor growth and metastasis [181]. Moreover, a dual relationship has been found between TGF-β and programmed cell death-1 (PD-1). For instance, PD-1 overexpression is associated with the induction of TGF-β, and TGF-β can regulate PD-1 expression [182,183]. This dual relationship is of importance in cancer therapy. Res can suppress the proliferation of oral cancer cells via the down-regulation of TGF-β and subsequent inhibition of PD-1. L-thyroxine as a thyroid hormone can also modulate the anti-tumor activity of Res via regulating the TGF-β/PD-1 axis [179].

Overall, the regulation of TGF-β by Res in cancer is of importance in terms of suppressing both migration and proliferation. The most well-known mechanism targeted by TGF-β is EMT, which can promote cancer metastasis. In addition, TGF-β can activate the signaling pathways such as PD-1 and Sox2 to ensure the growth and survival of cancer cells. Upon Res administration, TGF-β and its downstream targets are inhibited to pave the road for effective cancer therapy.

### 3.3. Resveratrol and Lung Injury

Injuries to vascular endothelium and alveolar epithelium by inflammatory factors can lead to the emergence of acute lung injury (ALI) [184]. Infections are able to generate ALI and among them, *Pseudomonas aerogenosa*, *Candidate albicans*, and *staphylococcal enterotoxin* B (SEB) are of importance [185,186,187]. In the amelioration of SEB-mediated lung injury, Res can target the TGF-β signaling pathway. Res can down-regulate the expression of miR-193a to inhibit TGF-β2 and TGFβR3, thus resulting in a decrease in levels of inflammatory cytokines and T cell infiltration [188]. The enhanced level of TGF-β has been associated with the development of asthma and lung injury [189]. In fact, the administration of Res may alleviate lung injury and asthma via decreasing levels of TGF-β [190]. Chronic obstructive pulmonary disease (COPD) is one of the most common disorders of lung tissue. Cigarette smoking is the most well-known reason for COPD [191]. Pulmonary inflammation, airflow obstruction, and remodeling are features of COPD [192]. Chronic inflammation can result in the development of COPD, and TGF-β has been found to play an important role in the pathogenesis of this disease [193,194]. Therefore, based on the modulatory impact of Res on TGF-β, the administration of this naturally occurring compound can be advantageous in the amelioration of COPD. It was also found that Res can decrease fibrotic response and inhibit mucus hypersecretion via the down-regulation of TGF-β [195].

It seems that via the regulation of TGF-β, Res is capable of reducing inflammation in lung and preventing the development of pathological events such as ALI, COPD, and asthma. Interestingly, Res inhibits inflammation via reducing the infiltration of cytokines and T cells. COPD is also emerged via pulmonary inflammation and fibrosis. Based on the effect of Res on TGF-β and subsequent decrease in fibrotic response and mucus hypersecretion, it can be beneficial in the treatment of COPD.

### 3.4. Resveratrol and Brain Injury

Cerebral hemorrhage is a leading cause of brain injury and vasospasm [196]. This malignancy results in ischemic/reperfusion and the induction of apoptosis in cancer cells [197,198]. The TGF-β signaling pathway has been correlated with brain injury [199]. Interestingly, the administration of Res was found to improve the blood–brain barrier (BBB) and inhibit apoptosis in neuronal cells. These protective effects of Res were found to be mediated via the inhibition of TGF-β-mediated ERK [200]. Moreover, it was found that exposing rats to alcohol is associated with an increase in levels of cytokines such as TGF-β. An administration of Res (10 and 20 mg/kg) can significantly improve cognitive deficits and reduces brain injury via decreasing TGF-β levels [201]. So, the alleviation of cognitive deficits and maintaining the integrity of BBB are functions of Res that can be mediated by TGF-β modulation.

### 3.5. Resveratrol and DM

**Figure 2:**
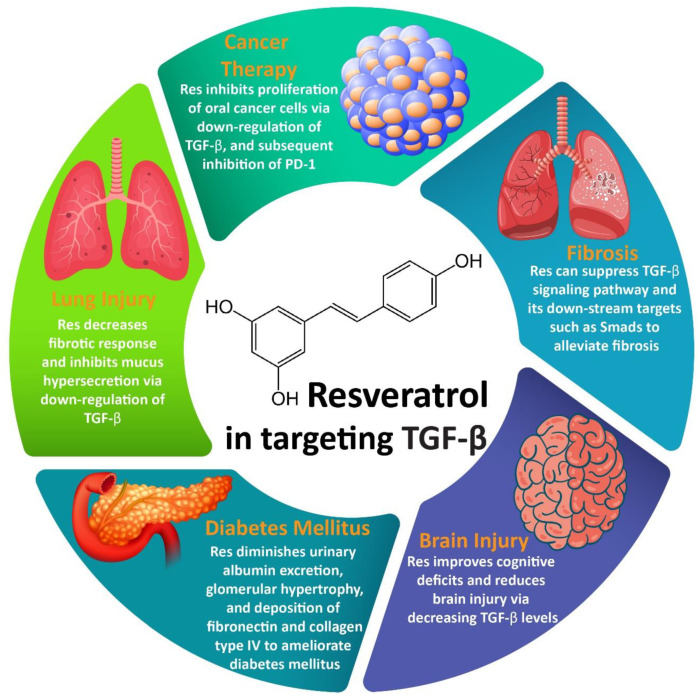
Regulation of TGF-β signaling by Res and its association with therapeutic effects.

During DM, microvascular complications can lead to hyperglycemia that accounts for the emergence of diabetic nephropathy (DN). Interestingly, an enhanced level of oxidative stress, renal polyol formation, protein kinase C induction, and activation of AMPK as well as the accumulation of advanced glycation end-products (AGEs) are responsible for DN [202,203]. TGF-β1 is considered as one of the potential pathways involved in the emergence of DN [204]. A combination of Res and rosuvastatin (RSU) was found to be beneficial in the alleviation of DN via the down-regulation of TGF-β1 [205]. The in vivo studies have also indicated that the administration of Res is a promising strategy in alleviating DN. It was observed that Res could diminish urinary albumin excretion, glomerular hypertrophy, and the deposition of fibronectin and collagen type IV to ameliorate DN. Moreover, an investigation of molecular pathways demonstrated that Res can alleviate TGF-β expression as well as the phosphorylation of Smad2 and Smad3 for DN alleviation (Table 1, Figure 2) [206]. The most important effect of Res during DN is reducing fibrosis, which can be mediated via TGF-β inhibition.

## 4. Conclusions and Future Directions

Currently, extensive research is being performed for possible applications of natural products for the therapy of chronic diseases, as these agents can regulate multiple molecular targets and transcription factors [226,227,228,229,230,231,232,233]. In the present review, a comprehensive discussion of possible impact of Res on the TGF-β signaling pathway, which is one of the important cascades involved in the regulation of biological mechanisms and the generation of pathological events, is provided. TGF-β acts as an upstream inducer of EMT, and this not only enhances the metastasis of cancer cells, but also mediates fibrosis in cells. Res inhibits TGF-β/EMT in suppressing both cancer and fibrosis. Through inhibiting TGF-β, Res diminishes the accumulation of collagen and fibrin, and reduces organ adhesion. Interestingly, Res dually targets both upstream (such as miRs) and downstream (Smads, PD-1, and EMT) mediators of TGF-β signaling in disease therapy. In addition to anti-tumor and anti-fibrotic activities, Res can also exert neuroprotective, lung protective, and anti-diabetic effects via the down-regulation of TGF-β, which was also highlighted in this article. Moreover, to circumvent the issue of poor bioavailability, the application of nanoparticles can enhance the modulatory effects of Res on the TGF-β signaling pathway. Besides, genetic manipulations such as small interfering RNA (siRNA) can also be co-applied for Res to promote its potential modulatory actions on TGF-β for therapeutic uses.

More studies are needed to find the optimal dose of Res in disease therapy via targeting TGF-β. Chemical modification of the Res structure and using nanoparticles can promote its efficacy in TGF-β regulation as well as its potential against various malignancies. More importantly, these findings are more valuable when they are translated into clinic. So, clinical studies are vital to approve the results of in vitro and in vivo experiments.

## Figures and Tables

**Table 1:** Res targets TGF-β signaling pathway in disease therapy.

Drug	In Vitro/In Vivo	Disease	Dose	Duration of Experiment	Administration Route	Effect on TGF-β	Results	References
Resveratrol Fenofibrate	In vivo (animal model of steatohepatitis)	Steatohepatitis	70 mg/kg	12 weeks	Diet	Inhibition	Alleviation of nonalcoholic steatohepatitis	[207]
Resveratrol	In vitro (rat mesangial cells) In vivo (rat model of diabetic nephropathy)	Diabetes	25 µM 20 mg/kg	24 h 4 weeks	Oral	Inhibition	Reducing mesangial cell viability, fibronectin secretion, and amelioration of diabetic nephropathy	[208]
Resveratrol	In vivo (diabetic mice)	Diabetes	5 and 25 mg/kg/day	2 months	Intragastric	Inhibition	Improving fibrosis via inhibition of ROS/ERK/TGF-β	[209]
Resveratrol	In vivo (diabetic rats)	Diabetes	10 mg/kg/day	30 days	Intraperitoneal	Inhibition	Alleviation of diabetic nephropathy and reducing epithelial desquamation, swelling, intracytoplasmic vacuolization, brush border loss, and peritubular infiltration	[210]
Resveratrol	In vivo (diabetic rats)	Diabetes	50 mg/kg	8 weeks	Gavage	Inhibition	Amelioration of renal damage and reducing collagen deposition	[211]
Resveratrol	In vivo (diabetic model)	Diabetes	10 mg/kg	8 weeks	Oral gavage	Inhibition	Reducing collagen deposition	[212]
Resveratrol	In vivo (diabetic rats)	Diabetes	10 mg/kg	4 weeks	Drinking water	Inhibition	Improving vascular dysfunction and reducing oxidative stress	[213]
Resveratrol	In vivo (rat model of chronic prostatitis)	Chronic prostatitis	10 mg/kg	10 days	Oral	Inhibition	Alleviation of prostate fibrosis via mast cell suppression	[214]
Resveratrol	In vivo (rat model of chronic prostatitis)	Chronic prostatitis	10 mg/kg	10 days	Oral	Inhibition	Reducing prostate fibrosis and urinary dysfunction via inhibition of TGF-β/Wnt/β-catenin	[215]
Resveratrol	In vitro (Human colorectal cancer cell line LoVo) In vivo (mice with orthotopic transplantation tumor)	Cancer	6 and 12 µM 50, 100, and 150 mg/kg	24 h 3 weeks	Intragastric	Inhibition	Suppressing metastasis of cancer cells by EMT inhibition via down-regulation of TGF-β/Smad signaling pathway	[216]
Resveratrol	In vitro (MCF-7 cells)	Cancer	5, 25, 50, 100, and 200 µM	48 h	-	Inhibition	Sensitizing cancer cells into chemotherapy via inhibition of TGF-β-mediated EMT	[217]
Resveratrol	In vitro (A431 human epidermoid carcinoma cells)	Cancer	50–100 µM	24 h	-	Inhibition	Suppressing ultraviolet-induced tumor proliferation	[218]
Resveratrol analogue (HS-1793)	In vivo (tumor bearing mice)	Cancer	0.5 and 1 mg/kg	3 weeks	Intraperitoneal	Inhibition	Enhancing efficacy of radiotherapy	[219]
Resveratrol	Murine model of LPS-induced pulmonary fibrosis	Pulmonary fibrosis	0.3 mg/kg	28 days	Intraperitoneal	Inhibition	Improving pulmonary fibrosis and inhibition of EMT via the down-regulation of TGF-β1/Smad	[216]
Resveratrol	In vivo (SIRT3-knock out mice)	Fibrosis	1.8 mg/kg	8 weeks	Diet	Inhibition	Improving cardiac fibrosis and suppressing fibroblast-to-myoblast transformation	[220]
Resveratrol	In vivo (chronic asthma model)	Asthma	10 and 50 mg/kg	3 months	Oral gavage	Inhibition	Inhibition of Smad2/3 phosphorylation, amelioration of airway inflammation and structural changes	[221]
Resveratrol	In vitro (human retinal pigment epithelial cells)	Eye disease	25, 50, 100, 200, 400, and 800 µM	24 h	-	Inhibition	Suppressing Smad2 and Smad3 phosphorylation leads to the inhibition of EMT and collagen deposition	[222]
Resveratrol	In vivo (mouse model of Duchene muscular dystrophy)	Muscular dystrophy	4 g/kg	32 weeks	Diet	Inhibition	Decreasing reactive oxygen species generation, fibronectin production, and enhancing expressions of α-SMA and SIRT1	[223]
Resveratrol	In vitro (rhabdomyosarcoma)	Rhabdomyosarcoma	5, 10, 20, 40, or 80 μmol/L	24, 48, and 72 h	-	Inhibition	Induction of G1 and S phases cell cycle arrest and down-regulation of Smad4	[224]
Resveratrol	In vivo (Male C57BL/6J mice)	-	5 mg/kg	2 days after surgery	Intraperitoneal	Inhibition	Reducing levels of collagen IV and fibronectin	[225]

TGF-β, transforming growth factor-beta; ROS, reactive oxygen species; ERK, extracellular signal-regulated kinase; EMT, epithelial-to-mesenchymal transition; α-SMA, α-smooth muscle actin, SIRT1, sirtuin 1.

## Abbreviations

NDs	neurological disorders
AD	Alzheimer’s disease
PD	Parkinson’s disease
TCM	Traditional Chinese Medicine
Res	resveratrol
NF-ĸB	nuclear factor-kappaB
IL	interleukin
TNF-α	tumor necrosis factor-α
WAT	white adipose tissue
BAT	brown adipose tissue
ABC	ATP binding cassette
Aβ	amyloid-beta
TGF-β	transforming growth factor-β
GI	gastrointestinal
CPC	centrifugal partition chromatography
BMPs	bone morphogenetic proteins
GDFs	growth and differentiation factors
LAP	latency associated peptide
PAI1	plasminogen activator inhibitor 1
EMT	epithelial-to-mesenchymal transition
SIRT7	sirtuin 7
DM	diabetes mellitus
MMP-9	matrix metalloproteinase-9
PF	pulmonary fibrosis
miR	microRNA
MF	myocardial fibrosis
RF	renal fibrosis
FMD	fibroblast-myofibroblast differentiation
TECs	tubular epithelial cells
SIRT1	sirtuin 1
PD-1	programmed cell death-1
ALI	acute lung injury
SEB	staphylococcal enterotoxin B
COPD	chronic obstructive pulmonary disease
BBB	blood-brain barrier
DN	diabetic nephropathy
ERK	extracellular signal-regulated kinase
MAPK	mitogen-activated protein kinase
AGEs	advanced glycation end-products
RSU	rosuvastatin
